# Changes in Anxiety Symptoms and Eating Patterns Among Emerging Adult Students in a Non-Traditional School Program During a Mathematics Examination

**DOI:** 10.3390/healthcare13202600

**Published:** 2025-10-15

**Authors:** Gustavo A. Hernandez-Fuentes, Laura A. Larios-Gomez, Jessica C. Romero-Michel, Kayim Pineda-Urbina, José M. Flores-Álvarez, Mario A. Corona-Arroyo, Daniel Tiburcio-Jiménez, Karmina Sánchez-Meza, Nomely S. Aurelien-Cabezas, Karen A. Mokay-Ramirez, Karla B. Carrazco-Peña, Alejandro Figueroa-Gutiérrez, Marina Delgado-Machuca, Iván Delgado-Enciso

**Affiliations:** 1 Department of Molecular Medicine, School of Medicine, University of Colima, Colima 28040, Mexico; ksmeza@ucol.mx (K.S.-M.); nomelyaurelien@gmail.com (N.S.A.-C.); dra_carrazco@ucol.mx (K.B.C.-P.); karla_machuca@ucol.mx (M.D.-M.); 2 Faculty of Chemical Sciences, University of Colima, Coquimatlan 28400, Mexico; llarios@ucol.mx (L.A.L.-G.); kpineda@ucol.mx (K.P.-U.); josemanuel@ucol.mx (J.M.F.-Á.); 3 State Cancerology Institute of Colima, Health Services of the Mexican Social Security Institute for Welfare (IMSS-BIENESTAR), Colima 28085, Mexico; ady_mokay@hotmail.com; 4 Faculty of Law, University of Colima, Colima 28040, Mexico; jessica_romero@ucol.mx; 5 Mining, Metallurgical and Geological Engineering Department, Engineering Division, Guanajuato University, Ex-Hacienda de San Matías S/N, Guanajuato 36020, Mexico; m.corona@ugto.mx; 6 Oficina Central Estatal de Los Servicios de Salud Para el Bienestar, Colima 28000, Mexico; daniel.tiburcio@imssbienestar.gob.mx; 7 Health Education Auxiliary Coordination, Mexican Institute of Social Security (IMSS), Villa de Alvarez 28984, Mexico; alejandro.figueroag@imss.gob.mx; 8 Department of Dietetics and Nutrition, Robert Stempel College of Public Health & Social Work, Florida International University (FIU-RCMI), Miami, FL 33199, USA

**Keywords:** academic stress, semi-school-based model, high school students, anxiety, food intake patterns

## Abstract

**Background/Objectives:** The semi-school-based model (SSBM) has gained momentum in Mexico post-COVID-19, providing flexibility for students managing work, family, and academics. However, little is known about how high-stakes academic evaluations affect the emotional well-being and lifestyle habits of students in this alternative setting. This study aims to assess two key research questions: (1) Does exposure to a high-stakes academic exam increase anxiety and depression levels in SSBM students? (2) Does exam-related stress affect dietary habits and physiological stress markers in these students? **Methods:** A prospective, longitudinal, quantitative study was conducted in September 2023 with 94 fourth-term high school students in an SSBM program in Mexico Assessments. Evaluations were conducted at two specific time points; Pre-exam Assessment Day (RCD): ~30–50 min before normal Saturday classes, and Exam-day Assessment (ED): ~30–50 min before the mathematics exam. Data included sociodemographic, HADS scores, dietary habits, and physiological measures (blood pressure, heart rate, oxygen saturation). Analyses were conducted in SPSS v28. Normality was tested using Kolmogorov–Smirnov. Paired continuous and categorical variables were compared with Wilcoxon signed-rank and McNemar’s or Fisher’s exact tests, respectively. Relative risks (RR) and multivariate logistic regression identified factors associated with anxiety and depression. Significance was set at *p* < 0.05. **Results:** Anxiety prevalence increased significantly on the exam day (from 22.3% to 59.6%, *p* < 0.001; RR = 14.281, 95% CI: 2.620–161.296), with no significant change in depression. Wilcoxon tests confirmed higher anxiety scores across both sexes. Systolic and diastolic blood pressure and heart rate increased significantly, particularly among females (*p* < 0.001), whereas oxygen saturation remained stable. Eating patterns shifted on ED, with higher consumption of fried foods, sweet bread, and sugar-sweetened beverages (*p* < 0.001), especially among males, and decreased intake of meat protein and vegetables, particularly among females. Meat consumption was significantly associated with increased anxiety (adjusted RR = 3.405; 95% CI: 1.035–11.194; *p* = 0.044). **Conclusions:** High-stakes academic exams in SSBM settings are associated with acute increases in anxiety and unhealthy dietary changes, even without significant depressive symptoms. These findings highlight the need for interventions supporting emotional regulation and healthy lifestyle behaviors among students facing academic stress in non-traditional educational environments.

## 1. Introduction

Education plays a pivotal role in shaping the life trajectories of individuals, with each stage of a student’s academic journey presenting unique challenges and transitions. One of the most critical periods is between the ages of 15 and 29—a phase marking the shift from adolescence to adulthood [1]. During this period, young people face combined pressures from education, work, and family responsibilities. In Mexico, these challenges are especially pronounced in smaller, less industrialized regions, where limited infrastructure and economic opportunities have historically led many to leave their pre-university or university studies in order to join the workforce [2,3].

Traditionally, educational systems have relied on full-time, weekday schedules where students attend classes continuously throughout the week. These models often emphasize standardized curricula, structured classroom instruction, and conventional assessment methods such as exams and continuous evaluations [4]. While these traditional systems are widely implemented and supported by educational policies and statistics, they may not adequately accommodate students who face additional responsibilities outside of school, such as work or family obligations, potentially limiting access and academic success for some populations [5].

In response to these circumstances, the past five years have seen the expansion of more flexible educational programs designed to accommodate students’ diverse needs. These programs offer adaptable schedules and reduced course loads, helping learners find a better balance between academic and personal responsibilities. A notable example is the semi-school-based model (SSBM), which refers to educational programs that depart from the traditional full-time, weekday schedule by offering flexible, often weekend-based or hybrid instruction designed for students who must reconcile academic progress with other significant life responsibilities. Students enrolled in SSBM are generally older than their counterparts in traditional educational systems, as these programs often attract individuals who are balancing education with work, family obligations, or other personal commitments. This trend is not limited to one region but is increasingly observed worldwide, as more students seek alternative educational paths that better align with their life circumstances [5].

To provide a broader perspective, it is important to consider several international examples of flexible or part-time learning models, including hybrid, weekend-based, or adaptive programs implemented in countries such as the United States, Germany, and Japan [6,7,8]. These programs, often referred to as immersive learning spaces, aim to accommodate students balancing education with work, family, or other personal responsibilities. Such programs share similarities with the SSBM, offering adaptable schedules and reduced course loads while maintaining academic rigor [9].

These flexible formats have gained traction not only in Mexico but also in various countries across Latin America and beyond, where structural inequalities and work-study tensions similarly limit educational access for young adults. Although not a new concept, the SSBM gained renewed relevance and experienced significant growth after the COVID-19 pandemic, as institutions around the world —including those in Mexico—sought to ensure educational continuity and increase accessibility [10]. Nevertheless, the implementation of these models also introduces new pedagogical and institutional challenges that require careful consideration [11].

Despite their growing relevance, non-traditional educational systems, like the SSBM, are often overlooked in educational planning and policymaking. Pedagogical strategies and institutional frameworks typically center around the traditional full-time student model, frequently neglecting the unique needs and experiences of students in SSBM or other alternative learning environments [12,13]. For instance, traditional education has long relied on exams as a primary tool for assessing learning outcomes even in the SSBM/Part time educational programs [14]. While recent reforms have promoted project-based learning to foster knowledge integration and real-world application, exams remain a dominant form of evaluation across diverse educational contexts.

However, limited research has examined how high-stakes assessments—particularly exams—affect students in non-traditional educational settings, especially in terms of their psychological and physiological responses. Most existing studies have focused on traditional educational models, where anxiety, poor nutrition, and inadequate rest are well-documented factors influencing academic performance. In these settings, the pressures associated with exams can have a significant impact on students’ mental and physical well-being. However, the dynamics in non-traditional educational environments, such as high school students enrolled in SSBM, may differ, and the magnitude of these effects could be more pronounced due to variations in structure, support, and student engagement [15,16].

In addition to these educational challenges, students in emergency-related professions or training programs have been reported to experience particularly high levels of stress, with anxiety and depression being among the most prevalent mental health conditions in this group [17]. The COVID-19 pandemic further amplified these issues, as prolonged uncertainty, social isolation, and the abrupt transition to remote learning led to an increase in psychological distress among students worldwide [18,19,20,21]. Furthermore, the academic years during the pandemic disrupted hands-on training and reduced opportunities for practical application of knowledge, limiting students’ professional preparedness in many disciplines [22]. Emerging evidence also suggests that the long-lasting consequences of the COVID-19 period include persistent symptoms of anxiety, depression, and academic disengagement, which may continue to affect students’ mental health and educational outcomes long after the return to in-person learning [23].

Considering this background, this study aims to investigate the relationship between physiological factors, lifestyle habits, and academic life in high school students enrolled in SSBM. Specifically, it will analyze the effect of academic evaluations—focusing on nutrition habits and anxiety levels—at two critical time points: the RCD and the ED of a standardized mathematics exam. In this context, the study seeks to explore how anxiety symptoms manifest in students during high-stakes examinations, what changes in eating patterns occur before and during these evaluations, and how the combined impact of non-traditional educational models together with post-pandemic challenges may influence students’ mental health and academic performance. The findings will contribute to a better understanding of the challenges faced by strategies to support their academic success and well-being. The results may also guide educational institutions in designing evidence-based interventions that align assessment practices with students’ holistic health needs.

## 2. Materials and Methods

### 2.1. Study Design

This study utilized a prospective, longitudinal, quantitative design with repeated measures, aimed at analyzing the relationship between physiological factors, lifestyle habits, and academic stress in high school students enrolled in a semi-school-based model (SSBM) with Saturday class schedules, during their fourth academic term. Evaluations were conducted at two specific time points—Pre-exam Assessment Day (RCD): ~30–50 min before normal Saturday classes, and Exam-day Assessment (ED): ~30–50 min before the mathematics exam. The study was carried out in September 2023 at a private high school in Colima, Mexico, approximately one year after the resumption of in-person educational activities following the COVID-19 pandemic. Both the structured interviews and physical examinations were conducted face-to-face by trained personnel.

The study population consisted of students in their fourth academic term at a private high school (equivalent to the last two years of secondary education) on a Saturday class schedule located in an urban area of Colima State, Mexico. Inclusion criteria were (1) enrollment in the Saturday fourth term of the SSBM high school program, (2) attendance on both RCD and ED, and (3) provision of verbal informed consent.

Exclusion criteria included students with previously diagnosed mental health disorders (e.g., generalized anxiety disorder, major depression), or uncontrolled or decompensated acute or chronic diseases that could interfere with physiological assessments, such as poorly controlled asthma, diabetes, or cardiovascular conditions. These criteria were applied to avoid confounding effects on physiological stress markers. Elimination criteria referred to participants who, after initial inclusion, (1) failed to complete all required instruments (e.g., missing one of the two evaluations or not completing the full questionnaire), (2) presented inconsistencies or errors in physiological data (e.g., measurement anomalies), or (3) voluntarily withdrew from the study [20,24]. The number of eliminated cases was minimal and represented less than 10% of the initial eligible group [25].

### 2.2. Data Collection and Instrument

Participants completed a questionnaire requiring approximately 10–15 min to answer. The questionnaire was divided into five sections, each focusing on different aspects of the participants’ lifestyle and health.

The first section collected demographic data, including gender, place of residence, and socioeconomic status. Socioeconomic status was categorized according to the 2022 guidelines of the Mexican Association of Market Intelligence and Opinion Agencies (AMAI), which classifies households into three groups: A/B (high), C (middle), and D/E (vulnerable middle class or low-income level) [2,26,27].

The second section focused on academic variables such as the number of failed subjects, regular class attendance, and general grade point average (GPA), which were used to assess students’ academic performance and engagement [28].

The third section assessed dietary habits using 12 items on a 4-point Likert scale (0 = never, up to 3 = very frequently), focusing on the type and frequency of food consumption, especially before academic sessions. This section was adapted from Martínez Coronado et al. (2023) [29].

The fourth section evaluated physical activity using the International Physical Activity Questionnaire-Short Form (IPAQ-SF), a validated tool for assessing the frequency, type, and duration of physical activity in adults [30]. For this study, the questionnaire was adapted to include specific questions on whether students engaged in physical activity before attending academic sessions.

The fifth section evaluated psychological health using the Spanish version of the Hospital Anxiety and Depression Scale (HADS), which consists of 11 items—six assessing depression and five assessing anxiety. Each item was rated on a 4-point Likert scale (0–3), with total scores ranging from 0 to 21 for each subscale. Cut-off points were set at ≥8 for anxiety and ≥7 for depression, based on validated thresholds. Participants were classified as either cases (with symptoms) or controls (without symptoms) [20,31]. For clarity, throughout this manuscript we will refer to these dimensions as “anxiety” and “depression”; however, it is important to note that the HADS does not provide a clinical diagnosis but rather identifies the presence and intensity of anxiety and depressive symptoms [20].

In addition to questionnaire data, behavioral variables such as wake-up time, transportation to school, travel time, history of allergies, and previous COVID-19 infection were collected. Health-related data included the presence of chronic conditions such as hypertension and diabetes [32,33]. Physiological parameters were measured at two points: RCD and ED. Oxygen saturation was assessed using a portable pulse oximeter (Servitae, Mexico City, Mexico), and blood pressure was measured with an automatic digital monitor (OMRON HEM-7121J, Kyoto, Japan) Normal oxygen saturation was defined as ≥95%, normal blood pressure as systolic 90–120 mmHg and diastolic 60–80 mmHg, and normal resting heart rate as 60–100 bpm for the Mexican population (based on local reference values). Values above or below these thresholds were used to classify physiological changes [24]. All measurements were conducted under standardized conditions—participants were seated and at rest for five minutes prior, and they refrained from food or caffeine for at least 30 min before testing, following international guidelines [34].

### 2.3. Ethics

All participants provided informed verbal consent, and the study was conducted in full compliance with international ethical standards, including the Declaration of Helsinki. Ethical considerations were strictly observed throughout the study, with an emphasis on maintaining data confidentiality in accordance with applicable data protection regulations to safeguard participants’ personal information. Furthermore, the study adhered to the STROBE guidelines for observational studies, ensuring transparent and comprehensive reporting of methods and results. The study was approved by the Research Ethics Committee of the State Cancer Institute and university authorities (approval number CEICANCL230317-ASEXCCC-06, 2 March 2017).

### 2.4. Statistical Analysis

Statistical analyses were performed using SPSS Statistics version 28 (IBM Corp., Armonk, NY, USA) [24]. Data normality was assessed using the Kolmogorov–Smirnov test, and as most variables were non-normally distributed, non-parametric tests were employed. These statistical tests were selected based on the type and distribution of the variables to accurately assess changes between the Pre-exam Assessment Day (RCD) and Exam-day Assessment (ED), ensuring robust and appropriate analysis of physiological, behavioral, and psychological measures.

Descriptive statistics: Continuous variables were expressed as medians with interquartile ranges (Q1–Q3), except for age (years), which was normally distributed and presented as mean with standard deviation (SD). Categorical variables were summarized as frequencies and percentages.

Inferential analyses were selected according to the type and distribution of the variables: Paired continuous variables [e.g., systolic and diastolic blood pressure, heart rate (defined as increase, decrease, or no change and categorized accordingly) and oxygen saturation] were compared using the Wilcoxon signed-rank test.

Paired categorical variables (e.g., consumption of specific foods, prevalence of anxiety or depression) were compared using McNemar’s test (Yes vs. No), or Fisher’s exact test as appropriate.

Relative risks (RR): For temporal comparisons between RCD and ED, crude relative risks (RR) with 95% confidence intervals (CI) were calculated using 2 × 2 contingency tables to evaluate the increase in anxiety (HADS ≥ 8) or depression (HADS ≥ 7) on the exam day. This approach captures temporal changes in the proportion of students experiencing clinically relevant anxiety or depression.

Adjusted odds ratios (aOR): To evaluate independent associations of behavioral and demographic factors with anxiety and depression during the exam day, binary logistic regression models were applied. Variables included in the models were selected based on theoretical relevance or observed differences (e.g., physical activity, diet, academic performance, age) [35,36]. Sex and age were not retained in the final models because they did not act as significant confounders (*p* = 0.482 and *p* = 0.444, respectively) [37,38]. Adjusted odds ratios (aOR) with 95% CI and *p*-values were reported [39].

Sample size was determined from the 103 students enrolled in the SSBM program. After applying exclusion criteria (incomplete participation in both assessments and history of psychological disorders), the final sample consisted of 94 students. This was considered adequate based on a sample size estimation using the ClinCalc tool, referencing the study by Olivia et al. (2022) [40,41], which reported a 20% increase in anxiety in a pre-post student evaluation. Under these assumptions, a minimum of 94 participant scores were required to achieve a power of 80% with an alpha level of 0.05 [42,43]. Subsequently, a post hoc statistical power analysis was performed following completion of the study, demonstrating that administration of the exam test increased the proportion of subjects exhibiting anxiety, resulting in a statistical power of 99.9% (α = 0.05). Limitations related to sample size and multivariate adjustments are discussed in the Discussion section, referencing prior publications that followed similar reasoning [44,45,46,47].

## 3. Results

### 3.1. Participant Selection and Demographics

**Figure 1:**
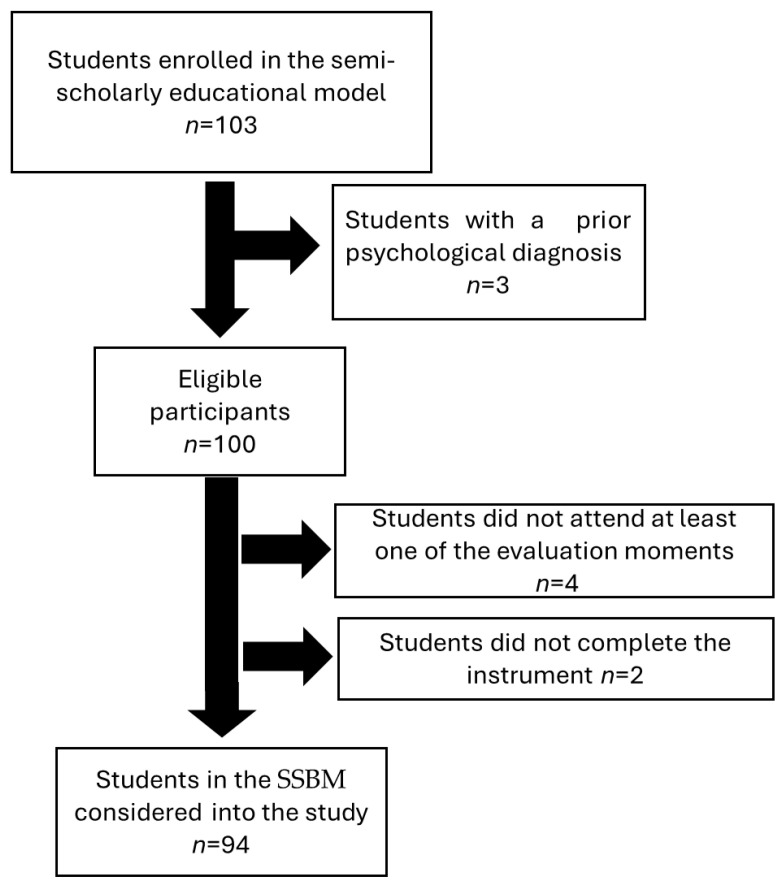
Participant Selection Flowchart: Invitation, Inclusion, and Exclusion.

From a total of 103 students enrolled in the SSBM program on fourth semester on Saturday schedule, 9 were excluded due to incomplete evaluations or pre-existing psychological diagnoses. Thus, the final analytical sample comprised 94 students. The participant selection process is summarized in Figure 1, which clearly distinguishes between those initially screened, those who met eligibility criteria, and those removed due to incomplete data.

Of the 94 students, 54.26% were male and 45.74% female, with a mean age of 20.95 ± 3.51 years (females: 20.72 ± 2.81; males: 21.14 ± 4.02). Most participants (53.19%) reported exercising occasionally, followed by daily (34.04%) and never (11.70%). Transportation methods varied, with walking (27.66%) being most common, followed by bus (17.02%), bicycle (14.89%), motorcycle (11.70%), and family car (7.45%). Regarding health status, 91.49% had no allergies and 92.55% had no chronic illnesses. Notably, 36.17% reported a prior COVID-19 infection, and 26.60% had failed at least one subject in the previous academic period, an aspect potentially relevant to academic stress. Full demographic, behavioral, and physiological baseline data are presented in Table 1.

### 3.2. Physiological Measurements RCD and ED of the Exam

All physiological measurements were performed under standardized conditions following established protocols to minimize external influences. As detailed in the Methods section, the first measurement was taken on RCD, and the second within 30 min on ED. Due to the non-normal distribution of most data (Shapiro–Wilk, *p* < 0.05), non-parametric tests were applied.

No significant changes were found in oxygen saturation between the RCD and ED. However, systolic and diastolic blood pressure increased significantly (*p* < 0.001), as did heart rate, suggesting heightened sympathetic activity and cardiovascular stress in anticipation of the exam. These effects were more pronounced in females, as detailed in Table 2.

### 3.3. Anxiety and Depression Symptoms: Prevalence and Risk Analysis

In addition to analyzing physiological changes, categorical analyses were conducted to assess the risk of developing clinically relevant anxiety or depression associated with exposure to exam-related stress. To do this, we used the Hospital Anxiety and Depression Scale (HADS), dichotomizing the scores based on established clinical thresholds: scores ≥ 8 for anxiety and ≥7 for depression were considered indicative of clinically significant symptoms.

The analyses focused on estimating the Relative Risk (RR) of developing anxiety or depression on the day of the exam. The RCD values were used as the reference group (non-exposed condition), and the day of the exam was considered the exposed group. This allowed us to examine the impact of exam stress on the likelihood of developing anxiety or depression, with the primary exposure being evaluated on the exam day.

Additionally, we considered gender and age as potential confounding variables in the analysis. However, the results showed that gender (*p* = 0.482) and age (*p* = 0.444) were not significant confounders, and therefore, they were not included in the final model.

Finally, the prevalence of anxiety and depression was calculated for both time points: the RCD and ED. These values were used to assess the relative risk of clinically relevant anxiety or depression associated with exposure to exam-related stress.

As shown in Table 3, a significant increase in the number of students meeting the criteria for anxiety was observed on exam day (from 21 cases [22.3%] to 56 cases [59.6%], *p* < 0.001), whereas the increase in depression cases was modest and statistically non-significant (from 34 cases [36.2%] to 45 cases [47.9%], *p* = 0.669).

We then conducted Relative Risk (RR) analyses, shown in Table 4, to quantify the association between exam-day exposure and clinically relevant anxiety or depression. The exposed group was defined as students evaluated on the day of the exam, and the outcomes were defined as scores above the clinical threshold for anxiety (≥8) or depression (≥7). No covariates were included in the final model, given the lack of significant confounding by sex or age. The RR was calculated using standard 2 × 2 contingency tables, comparing the proportion of cases in the exposed versus non-exposed groups.

The day of the exam (ED) was associated with a 14-fold increased risk of anxiety compared to the values obtained in RCD (RR = 14.281, 95% CI: 2.620–16.296, *p* < 0.001). No significant association was found for depression (RR = 0.789, 95% CI: 0.339–1.838, *p* = 0.583). No multivariate adjustment for sex, age, or other variables was performed.

**Figure 2:**
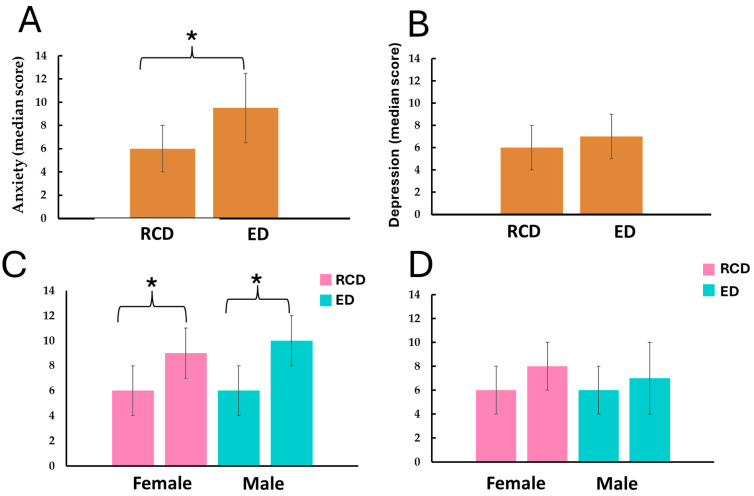
Anxiety and depression levels among students. Panels (**A**,**B**) show the overall levels for the entire sample, while Panels (**C**,**D**) display a breakdown by sex. Anxiety and depression were assessed using the Hospital Anxiety and Depression Scale (HADS), with clinical cut-off points of ≥8 for anxiety and ≥7 for depression. Data is presented as median (CI95%). * Statistical significance determined using the Wilcoxon test (*p* < 0.05). Pre-exam Assessment Day (RCD): ~30–50 min before normal Saturday classes and Ex-am-day Assessment (ED): ~30–50 min before the mathematics exam. Anxiety and depression were assessed using the Hospital Anxiety and Depression Scale (HADS). Values represent anxiety symptoms and depressive symptoms, not clinical diagnoses.

Figure 2 illustrates the changes in anxiety and depression levels, expressed as medians with Q1 and Q3. Anxiety increased significantly in the overall student population, rising from 6.0 (4.0–8.0) RCD to 9.5 (6.0–12.0) ED (*p* < 0.001). In contrast, depression showed no significant change, with medians of 6.0 (4.0–9.0) RCD and 7.0 (5.0–9.0) ED (*p* = 0.256).

When analyzed by sex, female students exhibited a significant increase in anxiety, from 6.0 (4.0–8.0) to 9.0 (7.0–12.0) (*p* < 0.001), while depression changed from 6.0 (3.0–10.0) to 8.0 (4.0–10.0) without statistical significance (*p* = 0.342). Male students also showed a significant increase in anxiety, from 6.0 (3.0–8.0) to 10.0 (6.0–12.0) (*p* < 0.001), with no significant change in depression (6.0 (3.0–8.0) to 7.0 (3.0–10.0), *p* = 0.542).

### 3.4. Differences in High-Calorie Food Consumption Between RCD (Pre-Exam Assessment) and ED (Exam-Day Assessment)

The analysis of lunch food consumption frequencies among high school students enrolled in an SSBM revealed significant shifts between Pre-exam Assessment Day (RCD): ~30–50 min before normal Saturday classes and Exam-day Assessment (ED): ~30–50 min before the standardized mathematics test (administered in the early afternoon) (Table 5). McNemar’s test was used to compare paired categorical data (Yes vs. No) between both days. Overall, students demonstrated a significant increase in the consumption of high-calorie foods—specifically fried items (*p* = 0.001), sweet bread (*p* = 0.001), and sugar-sweetened beverages (*p* = 0.004)—on the day of the exam. This shift was particularly notable among male students, who exhibited a significant increase in the consumption of sweet bread (*p* = 0.001) and sugar-sweetened beverages (*p* = 0.031). Female students also increased their intake of fried foods (*p* = 0.011) and sweet bread (*p* = 0.001), although the change in sugar-sweetened beverage consumption was not statistically significant (*p* = 0.089).

In contrast, the intake of healthier food options such as vegetables and fruits, meat protein, and eggs and cereals tended to decrease on the exam day. This was particularly evident in the general population for meat protein consumption, which significantly dropped (*p* = 0.013), and even more so among females (*p* = 0.001). While vegetable and fruit consumption decreased across all groups, the changes were not statistically significant. However, female students showed a trend toward preferring vegetables and cereal-based foods on regular school days, suggesting a more balanced diet prior to the test. These findings shows that academic stress or anticipation of the exam may influence students to choose more energy-dense, less nutritious foods, particularly among male students. This pattern highlights the importance of addressing dietary behaviors during high-stress academic periods to support better nutrition and potentially improve cognitive performance.

### 3.5. Dietary Influences on Anxiety and Depression: Temporal Analysis

Descriptive analyses using 2 × 2 tables and the calculation of relative risks (RR) allowed were performed to evaluate temporal changes in anxiety and depression symptoms from the Pre-exam Assessment Day (RCD) to the Exam-day Assessment (ED) (Table 6). This approach allowed the identification of preliminary associations between dietary factors and changes in symptom prevalence over the exam period. On the day before the exam, no dietary factor showed statistically significant associations with anxiety or depression. However, some trends, such as higher RR of anxiety among students consuming vegetables/fruits or sugar-sweetened beverages, suggest possible relationships that could be explored in studies with larger sample sizes. During the exam day, significant associations were observed: meat protein consumption was associated with an increased risk of anxiety (RR = 1.433; 95% CI: 0.979–2.096; *p* = 0.047), and sugar-sweetened beverage intake nearly doubled the risk of anxiety (RR = 1.985; 95% CI: 1.058–3.724; *p* = 0.008). Other dietary factors showed non-significant trends.

### 3.6. Multivariate Analysis of Dietary Factors Associated with Anxiety and Depression on ED

To evaluate independent associations, multivariate logistic regression was performed, producing adjusted odds ratios (aOR) (Table 7). This approach allows for the evaluation of independent associations after accounting for potential confounders. Notably, meat protein consumption at lunch was significantly associated with higher anxiety levels (Adjusted aOR = 3.405; 95% CI: 1.035–11.194; *p* = 0.044), suggesting a potential link between this dietary habit and emotional response under academic stress.

Although none of the other analyzed factors reached statistical significance in the adjusted model, some variables showed observable patterns. Sweet bread consumption showed an elevated, though non-significant, association with anxiety (aOR = 2.098; 95% CI: 0.781–5.639; *p* = 0.142). Meanwhile, the intake of vegetables/fruits, fried foods, eggs/milk/cereals, and sugar-sweetened beverages exhibited non-significant trends suggesting a possible protective effect (all *p* > 0.05). While these results are not conclusive, they highlight areas for further exploration. Future studies with larger sample sizes may help clarify these associations and provide greater statistical power to detect potential effects.

Regarding depression, no significant associations were found. Only physical activity before class and the intake of vegetables or sugar-sweetened beverages showed non-significant trends (all *p* > 0.05).

Other factors, such as prior failed subjects, age over 20 years, and other dietary components, showed no meaningful associations in the adjusted analysis. These findings highlight the potential role of certain dietary patterns, particularly meat consumption, in anxiety during academic stress and suggest avenues for future research into protective factors against depression in this context.

These multivariate analyses complement the descriptive RR findings by identifying factors independently associated with anxiety, while RR highlights raw temporal differences between RCD and ED.

## 4. Discussion

This study observed significant changes in anxiety and depression levels among high school students, with a slight upward trend in females. Before the exam (RCD), students, especially males, significantly increased their consumption of calorie-dense foods, including sweet bread, fried foods, and sugary drinks. Lunch consumption of animal-based proteins was associated with higher clinical anxiety, and a history of academic failure further increased the likelihood of anxiety during the exam. Taken together, these results provide a better understanding of academic, emotional, and behavioral factors interact in academic evaluation contexts and highlight the need for preventive strategies with a gender-sensitive approach and promotion of healthy habits.

While the study was not stratified by sex due to sample size limitations, a slight upward trend in female anxiety levels was observed. This observation aligns with previous studies reporting a higher prevalence of anxiety symptoms in similar academic evaluation contexts [48,49]. The greater impact on females may be related to psychosocial, hormonal, and gender socialization factors that predispose them to higher emotional reactivity to stress [50,51]. In this sense, our results are consistent with research reporting higher levels of state anxiety in students during school exams, even in contexts with low academic impact [52]. The association between previous academic failure and increased clinical anxiety reinforces the idea that negative past academic experiences heighten stress sensitivity during future evaluations, as has been documented in adolescents with a history of poor academic performance [53,54].

Changes in systolic and diastolic blood pressure on exam day reflected typical physiological responses to acute stress. This pattern has been described in studies simulating academic pressure situations, showing transient increases in cardiovascular activity attributable to activation of the hypothalamic–pituitary–adrenal axis and the sympathetic nervous system [55]. No gender differences were observed in this physiological response, which contrasts with studies reporting higher cardiovascular reactivity in males. Our findings suggest that the anticipation of the exam may have been enough to normalize the physiological response across genders [56], our findings may be due to the anticipation of the exam being sufficient to homogenize the physiological response among participants, regardless of gender.

In terms of eating habits, the rise in caloric and sugary food intake on exam day aligns with previous studies identifying food consumption as an emotional coping strategy [57]. This eating pattern, characterized by a higher preference for sweet and ultra-processed foods, has been described in university students during evaluation periods [58,59], although few studies document it in high school adolescents. The fact that animal protein consumption during lunch was associated with higher clinical anxiety levels is a novel finding and may be related to the nutritional quality of the food consumed or eating habits that reflect generally less healthy lifestyles. However, further studies are needed to clarify this relationship. In contrast, sweet bread consumption appeared to provide momentary emotional satisfaction, though it may have unfavorable metabolic implications over time [60].

While an association between animal-based protein intake and anxiety was observed, the causal direction remains uncertain. Anxiety may influence eating behaviors, leading individuals to seek comfort foods, including animal proteins, as part of emotional eating patterns. Conversely, some research has raised concerns about the long-term high consumption of red meat and processed foods, linking these dietary habits to inflammation, oxidative stress, and alterations in hormonal and neurotransmitter regulation, which may contribute to increased anxiety and cardiovascular risk [61]. These findings suggest a potential role in dietary patterns in modulating emotional responses during stressful situations such as exams. However, given the complex and possibly bidirectional nature of this relationship, further studies are needed to explore how diet, protein sources, and mental health interact, particularly among students facing academic stress.

It is important to note that this exploratory study was of short duration; nonetheless, it holds significant value for several reasons. First, SSBM systems, like the one evaluated, are often absent from discussions on educational policies in Mexico due to their lower student demand compared to traditional models, creating an information gap regarding the psychoemotional and health conditions of their students [62]. Second, it is relevant to consider that Mexico ranks among the highest in childhood and young adults’ obesity, a condition closely linked to an increased risk of metabolic syndrome and, in turn, has been widely associated with elevated anxiety and depression levels [63]. While it was not possible to record anthropometric measures in this study due to the characteristics of the environment, it is necessary to include them in future studies to explore the relationship between anxiety, eating habits, and metabolic risk. Nevertheless, it is recognized that this population may already have obesity-related issues or be on the path to developing them [64].

This study provides valuable insights by analyzing emotional, physiological, and dietary factors in students during a critical exam period. The findings highlight the complex interaction between academic pressure, emotional responses, and eating behaviors. While a slight tendency toward higher anxiety levels was observed among females, it was not possible to confirm significant gender differences in coping mechanisms due to sample size limitations. However, previous studies suggest that females may use coping strategies other than hypercaloric food intake, a hypothesis that warrants further investigation. In contrast, among male students, a clearer tendency toward emotional regulation through increased calorie intake was observed, consistent with reports on food-related coping strategies [65,66].

It is also important to consider that the relationship between anxiety and dietary patterns is likely bidirectional. On the one hand, certain dietary habits—such as high consumption of processed or calorie-dense foods—may influence emotional regulation and stress responses. On the other hand, anxiety itself may drive emotional eating behaviors, including a preference for comfort foods like sweets, fried foods, or meat-based products. Therefore, future studies should explore this complex interplay between diet, protein sources, and mental health, particularly among students in high-stress academic environments.

### Actual National Perspectives of SSBM and Limitations

Our findings highlight that, even within these flexible models, high-stakes exams continue to represent a significant stressor, influencing dietary habits and anxiety levels. Internationally, similar hybrid or weekend-based programs, such as those in Spain and Brazil, report comparable performance anxiety and somatic responses, suggesting that flexible learning environments, despite their pedagogical innovations, share vulnerabilities to stress-induced behavioral and psychological effects [67,68,69]. In Mexico, although there is still a research gap regarding the SSBM, many schools have implemented such flexible educational programs with minor variations in the application of standardized exams. These models often incorporate assessments that consider students’ collaborative work, soft skills, transversal and integrative project performance, as well as in-class engagement. Such approaches have been shown to improve students’ perception of learning and are adaptable to the specific contexts of each institution [70,71].

Regarding the limitations of the present study, given the unique nature of the SSBM, a relatively uncommon yet increasingly adopted format, the study had a limited number of participants. This constitutes a potential limitation for multivariate analyses. Nevertheless, the logistic regression model applied here can still be considered reliable under commonly accepted criteria. It has been postulated that at least ≤10 individuals with the event of interest per predictor variable are required in logistic regression models to improve model performance and reduce the risk of false-positive results [45,47]. In our multivariate logistic regression model (Table 5), eight predictor variables were included, analyzing 94 students with paired assessments. While this meets the general rule “at least 10 outcome events per predictor variable (EPV ≥ 10)”, it must be acknowledged that this guideline has been debated in the literature [44,46,72]. Therefore, the present findings should be interpreted with caution, and future studies with larger sample sizes are warranted to validate and strengthen these exploratory results.

Finally, these findings should be considered within the context of Mexican higher education, where high school dropout rates remain a significant concern [73]. According to recent data, only about 60% of students graduate from this educational level, a percentage that decreased further during the COVID-19 pandemic. This phenomenon has highlighted a decrease in student resilience, which calls for a rethinking of academic evaluation methods. Designing empathetic evaluation strategies that promote learning while minimizing psychological and nutritional harm is particularly important for vulnerable or marginalized populations. Therefore, the results of this study not only open the door for new research but should also be considered in the design of policies that integrate students’ emotional and physical well-being as a fundamental component of the educational process.

## 5. Conclusions

This study provides insights into how academic evaluations affect students’ emotional and physiological states in a semi-school-based, non-traditional educational setting a context rarely examined in research. Higher levels of anxiety symptoms, as measured by the HADS, and notable changes in physiological parameters, alongside shifts in dietary patterns, highlight the interplay of stress, coping strategies, and nutrition during exams. Students with higher intake of calorie-dense foods tended to report lower levels of anxiety symptoms, suggesting a potential coping mechanism. These findings emphasize the importance of designing supportive, gender-sensitive interventions and reconsidering standardized assessment methods to promote both emotional and physical well-being in students within new, non-traditional and underexplored educational models.

## Figures and Tables

**Table 1:** Demographic, Behavioral, and Physiological Characteristics of Students Evaluated.

Variable	Students (*n* = 94)
Sex	
Female	43 (45.74%)
Male	51 (54.26%)
Age (General, years)	20.95 ± 3.51
Age (Female, years)	20.72 ± 2.81
Age (Male, Years)	21.14 ± 4.02
Exercise frequency ^a^	
Daily	32 (34.04%)
Occasionally	51 (53.19%)
Never	11 (11.70%)
Number of failed academic subjects	2.50 ± 0.83
Regular class attendance	95.05 ± 5.5%
General grade point average ^b^	7.85 ± 1.23
Transportation mode	
Motorcycle	12 (11.70%)
Bus	16 (17.02%)
Taxi	4 (4.26%)
Family car	7 (7.45%)
Walking	24 (27.66%)
Bicycle	14 (14.89%)
Allergies	
No	86 (91.49%)
Yes	8 (8.51%)
Chronic illness	
No	87 (92.55%)
Yes	7 (7.45%)
COVID	
No	60 (63.83%)
Yes	34 (36.17%)
Failed subjects ^b^	
No	69 (73.40%)
Yes	25 (26.60%)

Data are presented as either *n* (%) or mean ± SEM, depending on the variable. The total sample included 94 participants from the fourth academic term (of a total of six). Percentages were calculated based on the total number of participants in each respective evaluation group. The variable “Failed subjects” refers to students who had failed at least one subject during the previous academic period. Regular class attendance refers to the percentage of in-person sessions attended. ^a^ Exercise frequency was categorized as: daily, occasional, or no exercise. ^b^ General grade point average was measured on a scale from 0 to 10.

**Table 2:** Physiological Parameters Measured on RCD and ED of the Exam Among High School Students in an SSBM.

Physiological Measurements	Evaluation		*p* Value *
	RCD Median (Q1–Q3)	ED Median (Q1–Q3)	
Oxygen saturation levels			
General	96.0 (76.0–99.0)	96.0 (83.0–99.0)	0.916
Female	97.0 (76.0–99.0)	97.0 (84.0–99.0)	0.465
Male	96.0 (83.0.0–99.0)	96.0 (83.0–99.0)	0.180
Systolic blood pressure			
General	116.0 (86.0–148.0)	130.0 (97.0–180.0)	<0.001
Female	113.0 (86.0–137.0)	120.0 (97.0–180.0)	<0.001
Male	120.0 (100.0–148.0)	136.0 (100.0–180.0)	<0.001
Diastolic blood pressure			
General	77.0 (55.0–109.0)	87.0 (60.0–112.0)	<0.001
Female	73.0 (55.0–109.0)	86.0 (70.0–99.0)	<0.001
Male	80.0 (56.0–102.0)	90.0 (60.0–112.0)	<0.001
Heart rate (beats per minute)			
General	74.0 (50.0–80.0)	94.0 (75.0–103.0)	<0.001
Female	76.0 (50.0–80.0)	96.0 (75.0–103.0)	<0.001
Male	78.0 (51.0–80.0)	98.0 (75.0–103.0)	<0.001

* Values are presented as median (Q1–Q3). Heart rate (beats per minute), oxygen saturation (%), and blood pressure (mmHg) were measured at two time points: Pre-exam Assessment Day (RCD): ~30–50 min before normal Saturday classes and Exam-day Assessment (ED): ~30–50 min before the mathematics exam. Comparisons were performed using Wilcoxon signed-rank test (*n* = 94). * *p* < 0.05 was considered statistically significant.

**Table 3:** Frequency of anxiety and depression on RCD and ED.

	RCD	ED	
Anxiety score ^a^	6 (4–8)	9.5 (6–12)	
Anxiety (score ≥ 8) ^b^	21 (22.3%)	56 (59.6%)	<0.001 *
Depression score ^a^	6 (3–9)	7 (4–10)	
Depression (score ≥ 7) ^b^	34 (36.2%)	45 (47.9%)	0.669 *

^a^ Number median (Q1–Q3) and ^b^ percentages of university students with clinically relevant anxiety (score ≥ 8) and depression (score ≥ 7) measured RCD and ED. *n* = 94. * Fisher’s exact test (*p* < 0.05). Pre-exam Assessment Day (RCD): ~30–50 min before normal Saturday classes and Exam-day Assessment (ED): ~30–50 min before the mathematics exam. Anxiety and depression were assessed using the Hospital Anxiety and Depression Scale (HADS). Values represent anxiety symptoms and depressive symptoms, not clinical diagnoses.

**Table 4:** Relative risk of developing anxiety and depression on ED compared to RCD.

Variable		95% Confidence Interval		*p*
	RR	Lower	High	
Anxiety	14.281	2.620	16.296	<0.001
Depression	0.789	0.339	1.838	0.583

Relative Risk (RR), 95% confidence intervals (CI), and *p*-values for factors associated with anxiety (levels ≥ 8) and depression (levels ≥ 7) in SSBM students. *p*-values were obtained using Chi-square tests. Anxiety and depression were assessed using the Hospital Anxiety and Depression Scale (HADS). Values represent anxiety symptoms and depressive symptoms, not clinical diagnoses.

**Table 5:** Frequency of Lunch Food Consumption by Sex and Evaluation Day in High School Students from an SSBM.

Variable	Evaluation Day				
	RCD (*n* = 94)		ED (*n* = 94)		*p* Value
Vegetables and fruits					
	Yes *n* (%)	No *n* (%)	Yes *n* (%)	No *n* (%)	
General	41 (43.6%)	53 (56.4%)	33 (35.1%)	61 (64.9%)	0.302
Male	19 (37.3%)	32 (62.7%)	18 (35.3%)	33 (64.7%)	1.000
Female	22 (51.2%)	21 (48.8%)	15 (34.9%)	28 (65.1%)	0.143
Eggs, milk and cereal					
	Yes *n* (%)	No *n* (%)	Yes *n* (%)	No *n* (%)	
General	59 (62.8%)	35 (37.2%)	55 (58.5%)	39 (41.5%)	0.677
Male	27 (52.9%)	24 (47.1%)	31 (60.8%)	20 (39.2%)	0.571
Female	32 (74.4%)	11 (25.6%)	24 (55.8%)	19 (44.2%)	0.152
Meat protein					
	Yes *n* (%)	No *n* (%)	Yes *n* (%)	No *n* (%)	
General	52 (55.3%)	42 (44.7%)	31 (33.0%)	63 (67.0%)	0.013
Male	23 (45.1%)	28 (54.9%)	20 (39.2%)	31 (60.8%)	0.742
Female	29 (67.4%)	14 (32.6%)	11 (26.6%)	32 (74.4%)	0.001
Fried foods					
	Yes *n* (%)	No *n* (%)	Yes *n* (%)	No *n* (%)	
General	44 (46.8%)	50 (53.2%)	76 (80.9%)	18 (19.1%)	0.001
Male	21 (41.2%)	30 (58.8%)	40 (78.4%)	11 (21.6%)	0.001
Female	23 (53.5%)	20 (46.5%)	36 (83.7%)	7 (16.3%)	0.011
Sweet bread					
	Yes *n* (%)	No *n* (%)	Yes *n* (%)	No *n* (%)	
General	10 (10.6%)	84 (89.4%)	24 (25.5%)	70 (74.5%)	0.001
Male	7 (13.7%)	44 (86.3%)	39 (76.5%)	12 (23.5%)	0.001
Female	7 (16.3%)	36 (83.7%)	15 (34.9%)	28 (65.1%)	0.001
Sugar-sweetened beverages					
	Yes *n* (%)	No *n* (%)	Yes *n* (%)	No *n* (%)	
General	29 (30.9%)	65 (69.1%)	51 (54.3%)	43 (45.7%)	0.004
Male	18 (35.3%)	33 (64.7%)	30 (58.8%)	21 (41.2%)	0.031
Female	11 (25.6%)	32 (74.4%)	21 (48.8%)	22 (51.2%)	0.089

Data are presented as frequency and percentage (*n*, %). The total sample consisted of 94 high school students evaluated RCD and ED of an academic exam. “General” refers to the combined total of male and female participants. “Meat protein” includes chicken, beef, or pork. The “Eggs and cereals” category refers to the consumption of eggs, oatmeal, whole grain cereal, cow’s milk, or oat milk. “Sugar-sweetened beverages“ include cola soft drinks, packaged juices, or nectar beverages. Exclusion criterion: Participants who did not complete both evaluations (RCD and ED) were excluded from the analysis. McNemar’s test was used to compare paired categorical data (Yes vs. No) between RCD and ED for each student. *p* < 0.05 was considered statistically significant. Pre-exam Assessment Day (RCD): ~30–50 min before normal Saturday classes and Ex-am-day Assessment (ED): ~30–50 min before the mathematics exam.

**Table 6:** Temporal associations between dietary factors and anxiety and depression (RCD → ED).

Variable	Anxiety RR (95% CI) RCD→ED	*p*	Depression RR (95% CI) RCD→ED	*p*
Physical activity prior to class	0.879 (0.632–1.224)	0.451	0.678 (0.447–1.028)	0.070
Vegetables and fruits	0.934 (0.650–1.342)	0.714	1.197 (0.719–1.994)	0.480
Eggs, milk and cereal	0.926 (0.640–1.340)	0.680	0.906 (0.535–1.478)	0.690
Meat protein consumption	1.433 (0.979–2.096)	0.047	0.776 (0.511–1.176)	0.237
Fried foods	0.459 (0.147–1.430)	0.137	1.007 (0.563–1.802)	0.980
Sweet bread	1.028 (0.702–1.506)	0.887	0.780 (0.446–1.367)	0.366
Sugar-sweetened beverages	1.985 (1.058–3.724)	0.008	1.041 (0.593–1.828)	0.888

RR = Relative Risk; CI = Confidence Interval; Anxiety and depression were assessed using the Hospital Anxiety and Depression Scale (HADS). Values represent symptoms, not clinical diagnoses. *p* = *p*-value from Chi-square test.

**Table 7:** Multivariate analysis of factors influencing ED.

Variable	Adjusted aOR	95% Confidence Interval		*p*
		Low	High	
Factors associated with anxiety				
Physical activity prior to class	1.296	0.408	4.116	0.660
Vegetables and fruits	0.884	0.280	2.794	0.834
Eggs, milk and cereal	0.837	0.292	2.396	0.740
Meat protein consumption	3.405	1.035	11.194	0.044
Fried foods	0.748	0.298	1.879	0.537
Sweet bread	2.098	0.781	5.639	0.142
Sugar-sweetened beverages	0.276	0.239	1.505	0.600
Subjects previously failed	1.151	0.379	3.489	0.804
Age > 20	0.723	0.291	1.793	0.483
Factors associated with depression				
Physical activity prior to class	2.418	0.616	9.487	0.206
Vegetables and fruits	0.551	0.185	1.642	0.284
Eggs, milk and cereal	1.343	0.445	4.059	0.601
Meat protein consumption	1.351	0.441	4.137	0.598
Fried foods	1.006	0.393	2.574	0.990
Sweet bread	0.975	0.362	2.625	0.960
Sugar-sweetened beverages	0.551	0.217	1.403	0.212
Subjects previously failed	0.775	0.250	2.400	0.659
Age > 20	1.281	0.511	3.211	0.598

The adjusted aOR (odds ratio) was calculated using multivariate binary logistic regression, including all listed variables in the model. A statistical model was generated to determine the factors associated with the test presentation (ED). Statistically significant *p* values are presented. Anxiety (≥8 points). Depression (≥7 points). Logistic regression model adjusted for all listed variables. Given the limited sample size (*n* = 94), results should be interpreted with caution. Anxiety and depression were assessed using the Hospital Anxiety and Depression Scale (HADS). Values represent anxiety symptoms and depressive symptoms, not clinical diagnoses.

## Data Availability

The data presented in this study are available on request from the corresponding author due to privacy, legal and ethical reasons.

## References

[1] Instituto Nacional de Estadística y Geografía (INEGI) (2020). Censo de Población y Vivienda. https://www.inegi.org.mx/programas/ccpv/2020/.

[2] Flores R., Telles E. (2012). Social Stratification in Mexico. Am. Sociol. Rev..

[3] Consejo Nacional de Población (CONAPO) (2022). La Situación Demográfica de México. https://www.gob.mx/conapo/documentos/la-situacion-demografica-de-mexico-2022.

[4] Reese L., Arauz R.M., Bazán A.R. (2011). Mexican Parents’ and Teachers’ Literacy Perspectives and Practices: Construction of Cultural Capital. Int. J. Qual. Stud. Educ..

[5] González Anaya A.G. (2020). La Disparidad Del Sistema Escolarizado y Semiescolarizado: Estudio de Egresados de La Carrera de Abogado. RIDE Rev. Iberoam. Para. Investig. Desarro. Educ..

[6] Ardissone A.N., Galindo S., Wysocki A.F., Triplett E.W., Drew J.C. (2021). The Need for Equitable Scholarship Criteria for Part-Time Students. Innov. High. Educ..

[7] Zaza S., Wright-De Agüero L.K., Briss P.A., Truman B.I., Hopkins D.P., Hennessy M.H., Sosin D.M., Anderson L., Carande-Kulis V.G., Teutsch S.M. (2000). Data Collection Instrument and Procedure for Systematic Reviews in the Guide to Community Preventive Services. Am. J. Prev. Med..

[8] Knopf J.A., Hahn R.A., Proia K.K., Truman B.I., Johnson R.L., Muntaner C., Fielding J.E., Jones C.P., Fullilove M.T., Hunt P.C. (2015). Out-of-School-Time Academic Programs to Improve School Achievement: A Community Guide Health Equity Systematic Review. J. Public. Health Manag. Pract..

[9] Villalobos López J.A. (2023). Los Posgrados En México En Modalidad No Escolarizada (Online) 2022. Rev. Paraguaya Educ. A Distancia (Reped).

[10] Schmelkes S. (2020). La Educación Superior Ante La Pandemia de La COVID-19: El Caso de México. Universidades.

[11] Wang X., Liu J., Jia S., Hou C., Jiao R., Yan Y., Ma T., Zhang Y., Liu Y., Wen H. (2024). Hybrid Teaching after COVID-19: Advantages, Challenges and Optimization Strategies. BMC Med. Educ..

[12] Cruz Vadillo R. (2020). Significados Sobre Política Educativa Desde La Perspectiva Del Profesorado de Educación Obligatoria y Superior En México. Rev. Educ..

[13] Alfaro-Ponce B., Alfaro-Ponce M., Muñoz-Ibáñez C.A., Durán-González R.E., Sanabria-Zepeda J.C., González-Gómez Z.L. (2023). Education in Mexico and Technological Public Policy for Developing Complex Thinking in the Digital Era: A Model for Technology Management. J. Innov. Knowl..

[14] García-Díaz R., Del Castillo E., Cabral R. (2020). Efficiency in Mexican Elementary Schools: A Regional Comparative. Investig. Econ..

[15] de Hoyos R., Estrada R., Vargas M.J. (2021). What Do Test Scores Really Capture? Evidence from a Large-Scale Student Assessment in Mexico. World Dev..

[16] Sokhanvar Z., Salehi K., Sokhanvar F. (2021). Advantages of Authentic Assessment for Improving the Learning Experience and Employability Skills of Higher Education Students: A Systematic Literature Review. Stud. Educ. Eval..

[17] Kelly D., Schroeder S., Leighton K. (2022). Anxiety, Depression, Stress, Burnout, and Professional Quality of Life among the Hospital Workforce during a Global Health Pandemic. J. Rural Health.

[18] Hernandez-Fuentes G.A., Barajas-Saucedo C.E. (2023). Estudiantes “Pre y Post Pandemia” Diferencias, Necesidades y Estrategias Empleadas En El Área de Ciencias Exactas y Experimentales. Nueva Presencialidad Escolar: Una Mirada Desde la Docencia.

[19] Hernandez-Fuentes G.A., Barajas-Saucedo C.E. (2022). Hábitos Alimenticios y Sus Alteraciones En Estudiantes En Confinamiento Por COVID-19. Textos Universitarios Sobre Cultura Física y Juventudes.

[20] Hernandez-Fuentes G.A., Romero-Michel J.C., Guzmán-Sandoval V.M., Diaz-Martinez J., Delgado-Enciso O.G., Garcia-Perez R.R., Godínez-Medina M., Zamora-Barajas V., Hilerio-Lopez A.G., Ceja-Espiritu G. (2024). Substance Use and Mental Health in Emerging Adult University Students Before, During, and After the COVID-19 Pandemic in Mexico: A Comparative Study. Diseases.

[21] Haikalis M., Doucette H., Meisel M.K., Birch K., Barnett N.P. (2022). Changes in College Student Anxiety and Depression from Pre-to During-COVID-19: Perceived Stress, Academic Challenges, Loneliness, and Positive Perceptions. Emerg. Adulthood.

[22] Jokar Z., Bijani M., Haghshenas H. (2025). Explaining the Challenges Faced by Nursing Students in Clinical Learning Environments during the Post-COVID Era: A Qualitative Content Analysis. BMC Res. Notes.

[23] Li F. (2022). Impact of COVID-19 on the Lives and Mental Health of Children and Adolescents. Front. Public Health.

[24] Dewitt B., Fischhoff B., Davis A.L., Broomell S.B., Roberts M.S., Hanmer J. (2019). Exclusion Criteria as Measurements I: Identifying Invalid Responses. Med. Decis. Mak..

[25] Schafer J.L., Graham J.W. (2002). Missing Data: Our View of the State of the Art. Psychol. Methods.

[26] Gerra G., Benedetti E., Resce G., Potente R., Cutilli A., Molinaro S. (2020). Socioeconomic Status, Parental Education, School Connectedness and Individual Socio-Cultural Resources in Vulnerability for Drug Use among Students. Int. J. Environ. Res. Public Health.

[27] Instituto Nacional de Estadística y Geografía (2020). Panorama Sociodemográfico de Colima Censo de Población y Vivienda.

[28] Al Hazaa K., Abdel-Salam A.-S.G., Ismail R., Johnson C., Al-Tameemi R.A.N., Romanowski M.H., BenSaid A., Rhouma M.B.H., Elatawneh A. (2021). The Effects of Attendance and High School GPA on Student Performance in First-Year Undergraduate Courses. Cogent Educ..

[29] Martínez Coronado A., Lazarevich I., Gutiérrez Tolentino R., Mejía Arias M.Á., Leija Alva G., Radilla Vázquez C.C. (2023). Construct Validity of a Questionnaire on Eating and Physical Activity Habits for Adolescents in Mexico City. Healthcare.

[30] Craig C.L., Marshall A.L., Sjöström M., Bauman A.E., Booth M.L., Ainsworth B.E., Pratt M., Ekelund U., Yngve A., Sallis J.F. (2003). International Physical Activity Questionnaire: 12-Country Reliability and Validity. Med. Sci. Sports Exerc..

[31] Barriguete Meléndez J.A., Pérez Bustinzar A.R., de la Vega Morales R.I., Barriguete Chávez-Peón P., Rojo Moreno L. (2017). Validation of the Hospital Anxiety and Depression Scale in Mexican Population with Eating Disorders. Rev. Mex. Trastor. Aliment..

[32] Negussie Y.M., Abebe A.T. (2025). Hypertension and Associated Factors among Patients with Diabetes Mellitus Attending a Follow-up Clinic in Central Ethiopia. Sci. Rep..

[33] Kalra S., Jena B., Yeravdekar R. (2018). Emotional and Psychological Needs of People with Diabetes. Indian J. Endocrinol. Metab..

[34] Muntner P., Einhorn P.T., Cushman W.C., Whelton P.K., Bello N.A., Drawz P.E., Green B.B., Jones D.W., Juraschek S.P., Margolis K.L. (2019). Blood Pressure Assessment in Adults in Clinical Practice and Clinic-Based Research. J. Am. Coll. Cardiol..

[35] Alcocer L., Chavez A., Gomez-Alvarez E., Espinosa C., Pombo J., Beaney T., Ster A.C., Poulter N.R. (2020). MMM18-Mexico investigators May Measurement Month 2018: An Analysis of Blood Pressure Screening Results from Mexico. Eur. Heart J. Suppl..

[36] Alcocer L., Palomo S., Rangel-Zertuche R.A., Berumen-Lechuga M.G., Medina-Serrano J.M., García-Cortés L.R., Mejía-Rodríguez O., Beaney T., Kerr G., Poulter N.R. (2024). May Measurement Month 2021: An Analysis of Blood Pressure Screening Results from Mexico. Eur. Heart J. Suppl..

[37] Midway S., Robertson M., Flinn S., Kaller M. (2020). Comparing Multiple Comparisons: Practical Guidance for Choosing the Best Multiple Comparisons Test. PeerJ.

[38] Rosner B. (2010). Fundamentals of Biostatistics.

[39] Sharma S.K., Mudgal S.K., Chaturvedi J. (2020). Expression and Interpretation of Relative Risk and Odds Ratio in Biomedical Research Studies. Indian J. Community Health.

[40] ClinCalc.com Statistics. Post-Hoc Power Calculator Post-Hoc Power Calculator. Evaluate Statistical Power of an Existing Study. https://clincalc.com/stats/Power.aspx.

[41] Kane S.P., Sample Size Calculator ClinCalc. https://clincalc.com/stats/samplesize.aspx.

[42] Sanjerehei M.M. (2023). Sample Size and Power Calculations for Nonparametric Tests in Vegetation Research. Theor. Appl. Ecol..

[43] Yin O., Parikka N., Ma A., Kreniske P., Mellins C.A. (2022). Persistent Anxiety among High School Students: Survey Results from the Second Year of the COVID Pandemic. PLoS ONE.

[44] van Smeden M., Moons K.G.M., de Groot J.A.H., Collins G.S., Altman D.G., Eijkemans M.J.C., Reitsma J.B. (2019). Sample Size for Binary Logistic Prediction Models: Beyond Events per Variable Criteria. Stat. Methods Med. Res..

[45] Lopez-Roblero A., Serrano-Guzmán E., Guerrero-Báez R.S., Delgado-Enciso I., Gómez-Manzo S., Aguilar-Fuentes J., Ovando-Garay V., Hernández-Ochoa B., Quezada-Cruz I.C., Lopez-Lopez N. (2024). Single-Nucleotide Polymorphisms in the Promoter of the Gene Encoding for C-Reactive Protein Associated with Acute Coronary Syndrome. Biomed. Rep..

[46] Van Smeden M., De Groot J.A.H., Moons K.G.M., Collins G.S., Altman D.G., Eijkemans M.J.C., Reitsma J.B. (2016). No Rationale for 1 Variable per 10 Events Criterion for Binary Logistic Regression Analysis. BMC Med. Res. Methodol..

[47] Shipe M.E., Deppen S.A., Farjah F., Grogan E.L. (2019). Developing Prediction Models for Clinical Use Using Logistic Regression: An Overview. J. Thorac. Dis..

[48] Agyapong-Opoku G., Agyapong B., Obuobi-Donkor G., Eboreime E. (2023). Depression and Anxiety among Undergraduate Health Science Students: A Scoping Review of the Literature. Behav. Sci..

[49] Chaplin T.M., Gillham J.E., Seligman M.E.P. (2009). Gender, Anxiety, and Depressive Symptoms. J. Early Adolesc..

[50] Verma R., Balhara Y.S., Gupta C. (2011). Gender Differences in Stress Response: Role of Developmental and Biological Determinants. Ind. Psychiatry J..

[51] Farhane-Medina N.Z., Luque B., Tabernero C., Castillo-Mayén R. (2022). Factors Associated with Gender and Sex Differences in Anxiety Prevalence and Comorbidity: A Systematic Review. Sci. Prog..

[52] Yarkwah C., Kpotosu C.K., Gbormittah D. (2024). Effect of Test Anxiety on Students’ Academic Performance in Mathematics at the Senior High School Level. Discov. Educ..

[53] Downing V.R., Cooper K.M., Cala J.M., Gin L.E., Brownell S.E. (2020). Fear of Negative Evaluation and Student Anxiety in Community College Active-Learning Science Courses. CBE—Life Sci. Educ..

[54] England B.J., Brigati J.R., Schussler E.E., Chen M.M. (2019). Student Anxiety and Perception of Difficulty Impact Performance and Persistence in Introductory Biology Courses. CBE—Life Sci. Educ..

[55] Mak H.W., Gordon A.M., Prather A.A., Epel E.S., Mendes W.B. (2023). Acute and Chronic Stress Associations with Blood Pressure: An Ecological Momentary Assessment Study on an App-Based Platform. Psychosom. Med..

[56] Bouakkar J., Pereira T.J., Johnston H., Pakosh M., Drake J.D.M., Edgell H. (2024). Sex Differences in the Physiological Responses to Cardiac Rehabilitation: A Systematic Review. BMC Sports Sci. Med. Rehabil..

[57] Jacques A., Chaaya N., Beecher K., Ali S.A., Belmer A., Bartlett S. (2019). The Impact of Sugar Consumption on Stress Driven, Emotional and Addictive Behaviors. Neurosci. Biobehav. Rev..

[58] Durán-Agüero S., Valdés-Badilla P., Valladares M., Espinoza V., Mena F., Oñate G., Fernandez M., Godoy-Cumillaf A., Crovetto M. (2023). Consumption of Ultra-Processed Food and Its Association with Obesity in Chilean University Students: A Multi-Center Study. J. Am. Coll. Health.

[59] Widłak P., Malara M., Tomczyk Ł., Dania A., Panagiotakou G., Papoulia G. (2024). Evaluation of the Dietary Habits of Polish and Greek University Students in the Context of the Health Benefits of Their Diets. Nutrients.

[60] McCrickerd K., Lensing N., Yeomans M.R. (2015). The Impact of Food and Beverage Characteristics on Expectations of Satiation, Satiety and Thirst. Food Qual. Prefer..

[61] de Souza F.D., Fidale T.M., Pereira T.C.R., Mantovani M.M., Deconte S.R., Moreira-Silva D., de Moura F.B.R., Martins L.D.Q., Alex dos Santos L., da Silva Medeiros  R. (2023). Effects of Hyperprotein Diet on Anxiety, Haemodynamics and Morphofunctional Aspects of the Heart of Wistar Rats. Exp. Physiol..

[62] Islas P.M., Calef A.K., Aparicio C. (2021). 2013 Mexico’s Education Reform: A Multi-Dimensional Analysis. Implementing Deeper Learning and 21st Education Reforms.

[63] Gutiérrez Tolentino R., Lazarevich I., Gómez Martínez M.A., Barriguete Meléndez J.A., Schettino Bermúdez B., Pérez González J.J., del Muro Delgado R., Radilla Vázquez C.C. (2024). Epidemiological Overview of Overweight and Obesity Related to Eating Habits, Physical Activity and the Concurrent Presence of Depression and Anxiety in Adolescents from High Schools in Mexico City: A Cross-Sectional Study. Healthcare.

[64] Padilla C.J., Ferreyro F.A., Arnold W.D. (2021). Anthropometry as a Readily Accessible Health Assessment of Older Adults. Exp. Gerontol..

[65] Robert M., Shankland R., Bellicha A., Kesse-Guyot E., Deschasaux-Tanguy M., Andreeva V.A., Srour B., Hercberg S., Touvier M., Leys C. (2022). Associations between Resilience and Food Intake Are Mediated by Emotional Eating in the NutriNet-Santé Study. J. Nutr..

[66] Ljubičić M., Matek Sarić M., Klarin I., Rumbak I., Colić Barić I., Ranilović J., Dželalija B., Sarić A., Nakić D., Djekic I. (2023). Emotions and Food Consumption: Emotional Eating Behavior in a European Population. Foods.

[67] Singh J., Evans E., Reed A., Karch L., Qualey K., Singh L., Wiersma H. (2022). Online, Hybrid, and Face-to-Face Learning Through the Eyes of Faculty, Students, Administrators, and Instructional Designers: Lessons Learned and Directions for the Post-Vaccine and Post-Pandemic/COVID-19 World. J. Educ. Technol. Syst..

[68] Robinson J., Banerjee I., Reechaye D., Perrine A.L.A. (2025). Medical Students’ Perspectives on Physical, Online, and Hybrid Learning Modalities: A Mixed Methods Study from a Medical School in Mauritius. Cureus.

[69] van den Beuken M., Loos I., Maas E., Stunt J., Kuijper L. (2025). Experiences of Soft Skills Development and Assessment by Health Sciences Students and Teachers: A Qualitative Study. BMC Med. Educ..

[70] Sánchez Ambriz G., Yáñez Hernández A., Sánchez Ambriz M.L. (2022). Talento Humano En Las Organizaciones: Competencias Blandas Para La Empleabilidad de Estudiantes Universitarios. Rev. Univ. Digit. Cienc. Soc. (RUDICS).

[71] Chaves-Oviedo M.L., Dorado-Martinez A.D. (2019). Desarrollo de Habilidades Para La Vida En Escuela Móvil: Estrategia de Empoderamiento de Niños y Adolescentes Como Sujetos de Derecho. Univ. Salud.

[72] Peduzzi P., Concato J., Kemper E., Holford T.R., Feinstem A.R. (1996). A Simulation Study of the Number of Events per Variable in Logistic Regression Analysis. J. Clin. Epidemiol..

[73] Juárez U., Cetina N., Victoria C., Veniamin V., Sánchez M., Maribel M., Argente V., Alberto R., Martínez P., Mariana K. (2023). Student Dropout in Higher Education in Southern Mexico: A Qualitative Approach (2011–2020). Emerg. Trends Educ..

